# Cu(II) and magnetite nanoparticles decorated melamine-functionalized chitosan: A synergistic multifunctional catalyst for sustainable cascade oxidation of benzyl alcohols/Knoevenagel condensation

**DOI:** 10.1038/s41598-019-53765-3

**Published:** 2019-11-28

**Authors:** Zahra Alirezvani, Mohammad G. Dekamin, Ehsan Valiey

**Affiliations:** Pharmaceutical and Heterocyclic Compounds Research Laboratory, Department of Chemistry, Iran University of Science and Technology, Tehran, 1684613114 Iran

**Keywords:** Chemistry, Materials science, Nanoscience and technology, Environmental chemistry

## Abstract

The uniform decoration of Cu(II) species and magnetic nanoparticles on the melamine-functionalized chitosan afforded a new supramolecular biopolymeric nanocomposite (Cs-Pr-Me-Cu(II)-Fe_3_O_4_). The morphology, structure, and catalytic activity of the Cs-Pr-Me-Cu(II)-Fe_3_O_4_ nanocomposite have been systematically investigated. It was found that Cs-Pr-Me-Cu(II)-Fe_3_O_4_ nanocomposite can smoothly promote environmentally benign oxidation of different benzyl alcohol derivatives by *tert*-butyl hydroperoxide (TBHP) to their corresponding benzaldehydes and subsequent Knoevenagel condensation with malononitrile, as a multifunctional catalyst. Interestingly, Fe_3_O_4_ nanoparticles enhance the catalytic activity of Cu(II) species. The corresponding benzylidenemalononitriles were formed in high to excellent yields at ambient pressure and temperature. The heterogeneous Cs-Pr-Me-Cu(II)-Fe_3_O_4_ catalyst was also very stable with almost no leaching of the Cu(II) species into the reaction medium and could be easily recovered by an external magnet. The recycled Cs-Pr-Me-Cu(II)-Fe_3_O_4_ was reused at least four times with slight loss of its activity. This is a successful example of the combination of chemo- and bio-drived materials catalysis for mimicing biocatalysis as well as sustainable and one pot multistep synthesis.

## Introduction

Nanoparticles (NPs) have attracted great attention because of their unique optical, electrochemical, medical and catalytic properties^1–8^. Indeed, the capacities and applications of NPs, as sorbent, sensor, heterogeneous catalyst, etc, are limited due to their low sustainability and dispersibility^9–11^, deactivation or constant leaching, and low recyclability^12^. Fortunately, the properties of NPs are affected seriously by support materials such as inorganic porous materials mainly silica or alumina^13^, organic polymers or biopolymers^14,15^, dendrimers^16^, carbon nanotubes^17^, graphene oxide^18^, etc., which are essential for their applications specially in heterogeneous catalysis^19–21^. Because of natural abundance, low toxicity compared to other transition metals and redox potential, copper is an appropriate metal used in nature for many oxidation reactions^22–24^. Therefore, the immobilization of Cu(II) and magnetite NPs on multifunctional group supports can be considered as a feasible way to address above mentioned problems and sustainable chemistry principles^11,25^. In this way, supramolecular chelation of Cu(II) species onto the surface of bifunctional modified chitosan with enhanced catalytic activity would be very desirable. This is a successful example of the combination of chemo- and bio-drived materials catalysis for mimicing biocatalysis as well as sustainable and one pot multistep synthesis^26–32^. Indeed, one-pot multistep reactions which are also known as cascade reactions, do not require the isolation of intermediates and reduce the solvent wastes. Hence, cascade reactions have attracted a great deal of attention in both academia and industry in recent decades^29,33–36^. These reactions afford desired products in high to excellent yeilds and hence properly adress green and sustainable chemistry principles. To date, most of the reported catalysts for one-pot cascade reactions are homogeneous, which typically suffer from product pollution and poor recyclability. Therefore, designing and introducing multifunctional catalysts with different catalytically active sites for one-pot cascade reactions is still a serious challenge.

**Scheme 1:**
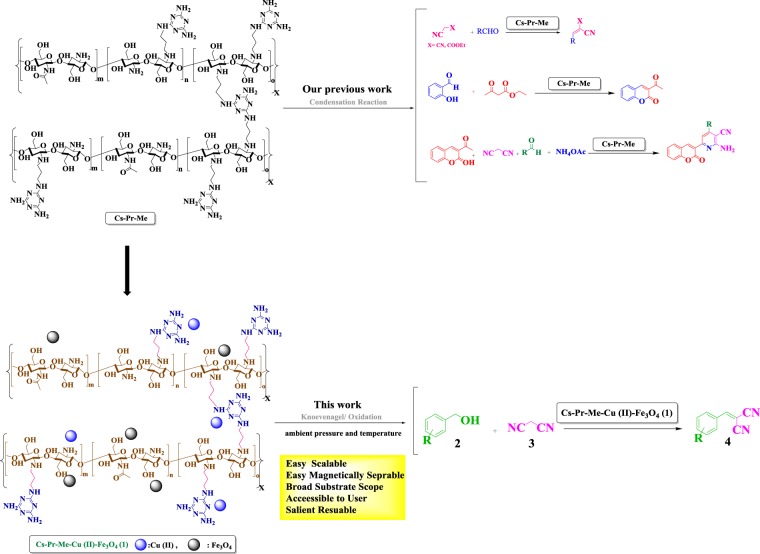
Oxidation/Knoevenagel condensation of different benzyl alcohol derivatives with malononitrile in the presence of the Cs-Pr-Me-Cu(II)-Fe_3_O_4_ (**1**).

Cyanocinamonitriles, which are prepared by the Knoevenagel condensation of a carbonyl group and C–H acids, serve as key intermediates in the synthesis of many important fine chemicals^37–42^. Nowadays, the tandem oxidation of alcohols and Knoevenagel condensation reaction of the obtained aldehydes and active methylene compounds, as an economic and eco-friendly procedure towards cyanocinamonitriles, has also received a great attention from green and sustainable stand points of view^35,43–47^. Traditionally, stoichiometric amounts of oxidants such as permanganates^48^, chromium reagents^49^ or the Dess-Martin periodinane^40^ have been used for oxidation reactions with significant environmental impacts due to their toxicity and producing large amounts of waste. To address the aformentioned concerns, we were interested to modify chitosan, as a naturally abundant biopolymer with suitable ligands to afford multiple active sites^50^. In the previous repots, we succeeded in covalentlly modification of chitosan with melamine and exploring of the activity of obtained Cs-Pr-Me as a bifunctional bio-derived organocatalyst^51,52^. To our delight, the Cs-Pr-Me materials, with plentiful amino and hydroxyl groups in a proper geometry, was found to be a suitable support for the immobilization of Cu(II) and magnetic nanoparticles. Due to the economic and sustainable benefits of magnetic copper-based catalysts^25^ and in continuation of our ongoing efforts towards developing efficient and novel heterogeneous catalysts^15,51–56^, we wish herein to report preparation and characterization of the supramolecular Cu(II) and magnetite nanoparticles decorated melamine-functionalized chitosan (Cs-Pr-Me-Cu (II)-Fe_3_O_4_), as a recyclable catalyst, for green cascade oxidation/Knoevenagel condensation. Furthermore, the use of TBHP, as a green oxidant, is a reasonable choice^55^ (Scheme 1).

## Results and Discussion

The Cs-Pr-Me materials were prepared by grafting of melamine to the chitosan backbone using 1,3-dibromopropne, as an appropriate linker, according to our published procedures^51,52^. In the next step, immobilization of the Cu(II) species on the Cs-Pr-Me backbone was achieved using an aqueous solution of Cu(OAc)_2_. Then, the surface of Cs-Pr-Me were decorated ***in situ*** by magnetite nanoparticles. The obtained Cs-Pr-Me-Cu(II)-Fe_3_O_4_ materials were characterized in details with various analysis techniques and methods such as Fourier transform infrared (FTIR) spectroscopy, field emission scanning electron microscopy (FESEM), transmission electron microscopy (TEM), inductively coupled plasma (ICP), X-ray diffraction (XRD), energy dispersive X-ray (EDX) mapping analysis, and vibrating sample magnetometer (VSM).

The FTIR spectra of Cs-Pr-Me-Cu(II)-Fe_3_O_4_ (**1**) demonstrated the evidence for existence of Cu(II) and magnetite nanoparticles on the melamine-functionalized chitosan backbone. Fig. S1 illustrates the FTIR spectra of Cs-Pr-Me (a), fresh Cs-Pr-Me-Cu(II)-Fe_3_O_4_ (**1**, b) and Cs-Pr-Me-Cu(II)-Fe_3_O_4_ after four times recycling (**1**, c), respectively (See Electronic Supplementary Information). As shown in Fig. S1a, the Cs-Pr-Me showed the adsorbtion bands at 3567, 3453, 3450–3100 and 1390 cm^−1^, which are attributed to the asymmetric and symmetric stretching vibrations of N–H bonds and the stretching vibration of O–H bonds of chitosan. Furthermore, the signals at 1616 and 1593 cm^−1^ are assigned to the NH_2_ and NCN bending vibrations of melamine. The OH bending vibration of chitosan corresponds to the broad signal in the range of 1470–1330 cm^−1^. After formation of Cs-Pr-Me-Cu(II)-Fe_3_O_4_ (**1**), the above band intensities were decreased as shown in Fig. S1b, which represent Cu(II) and magnetite nanoparticles have been coordinated by the amino and hydroxyl functional groups of the Cs-Pr-Me materials. In addition, the characteristic bands for Fe-O and Cu-O stretching vibrations were observed at 560 and 430 cm^−1^, respectively.

**Figure 1:**
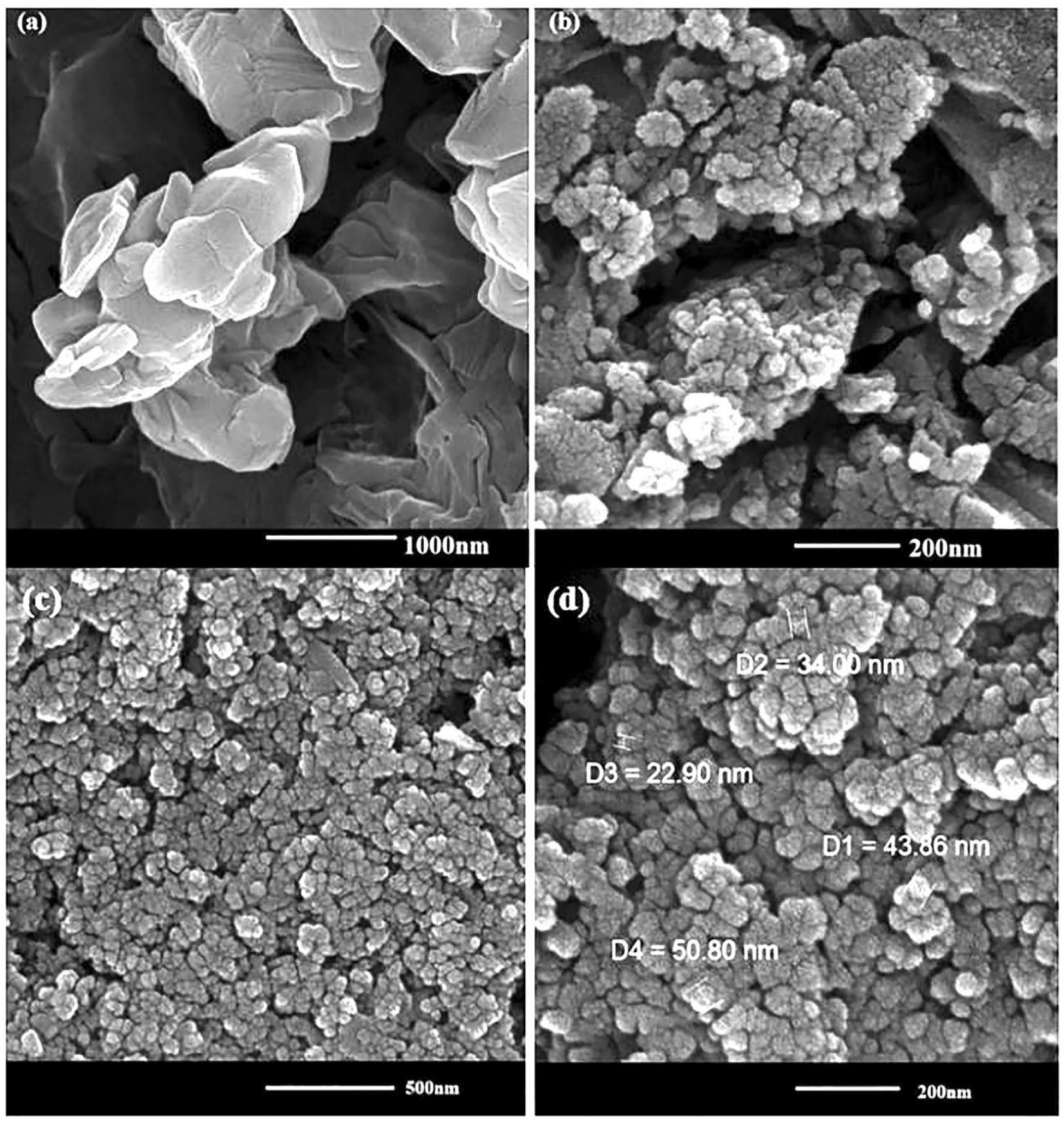
FESEM images of the commercial chitosan (**a**), Cs-Pr-Me materials befor modification (**b**), and Cs-Pr-Me-Cu(II)-Fe_3_O_4_ (**1**) materials at 500 (**c**) and 200 nm (**d**) scales.

**Figure 2:**
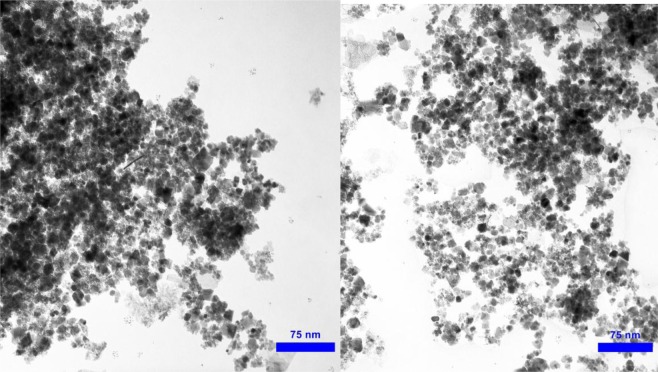
TEM images of the Cs-Pr-Me-Cu(II)-Fe_3_O_4_ (**1**) nanomaterials.

**Figure 3:**
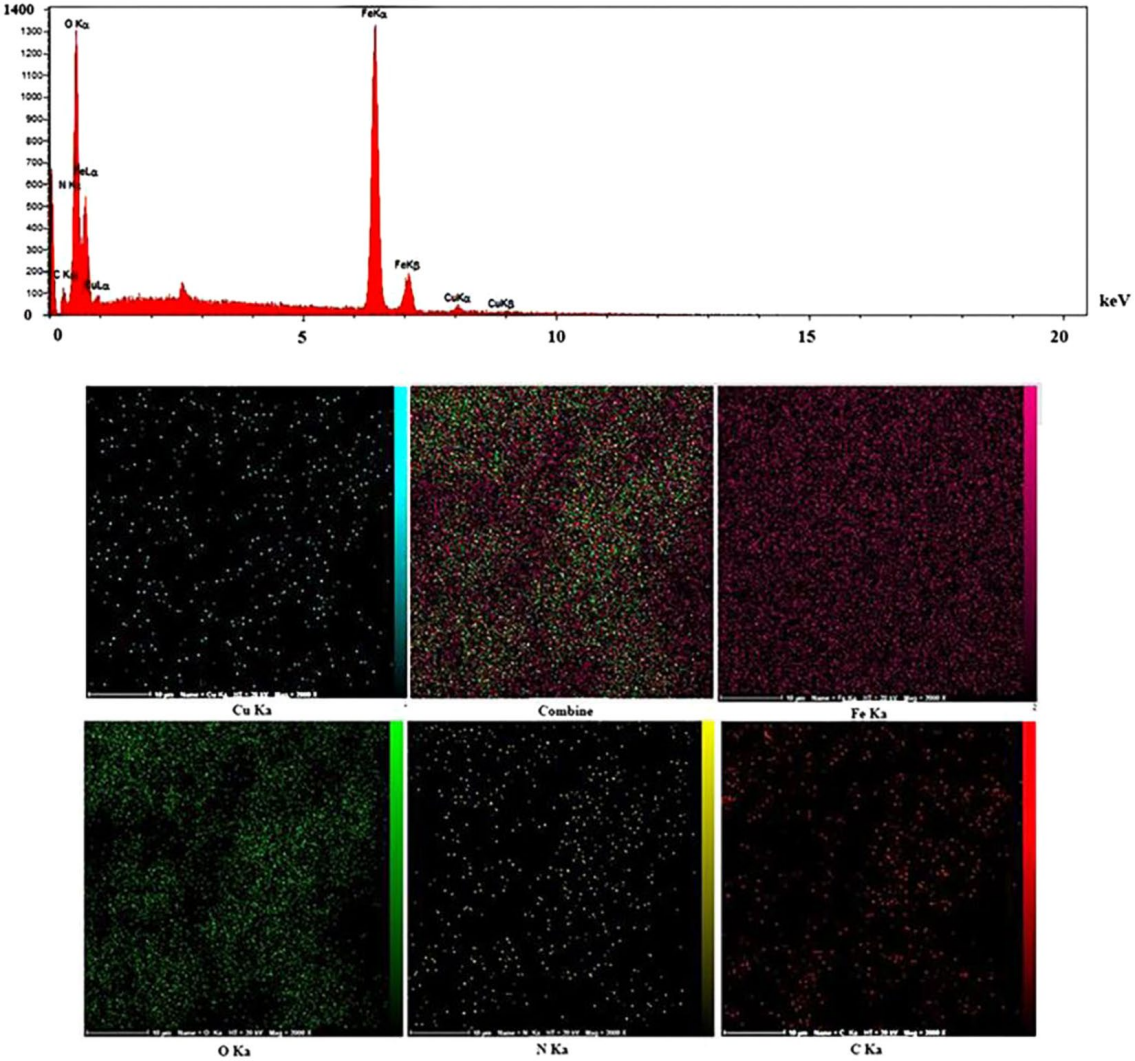
Energy dispersive spectroscopy (EDX) pattern and elemental mapping of the Cs-Pr-Me-Cu(II)-Fe_3_O_4_ (**1**) nanocomposite.

The morphological features and size of Cu(II) and magnetic iron oxide NPs were examined by FESEM and TEM experiments (Figs. 1 and 2). The FESEM images of the commercial chitosan and Cs-Pr-Me befor modification have been shown in Fig. 1a,b, respectively. Furtthermore, Fig. 1c,d show the FESEM images of Cs-Pr-Me-Cu(II)-Fe_3_O_4_ (**1**) materials. The Cu(II) and Fe_3_O_4_ nanoparticles are approximately spherical and scattered with an average diameter of about 23–51 nm. On the other hand, TEM images (Fig. 2) obviously demonstrate almost uniform decoration of Cu(II) species and magnetic nanoparticles on the melamine-functionalized chitosan support. Also, according to ICP analysis, the percentage of chelated copper and iron in the fresh Cs-Pr-Me-Cu(II)-Fe_3_O_4_ (**1**) was found to be 1.38 wt% (Cu) and 25.72 wt% (Fe), respectively. The EDX spectrum of the Cs-Pr-Me-Cu(II)-Fe_3_O_4_ (**1**) is shown in Fig. 3. The EDX spectrum indicates that the introduced catalyst **1** materials are composed of Cu, Fe, O, N and C elements. In addition, EDX mapping was performed to observe distributions of the elements in the Cs-Pr-Me-Cu(II)-Fe_3_O_4_ (**1**) nanocomposite. As it can be seen in Fig. 3, the indicated elements, especially Cu and Fe, demonstrate uniform distributions. These obtained data strongly confirm successful immobilization of the Cu(II) species and iron oxide nanoparticles on the Cs-Pr-Me backbone. On the other hand, the magnetic properties of the Cs-Pr-Me-Cu(II)-Fe_3_O_4_ (**1**) was measured using vibrating sample magnetometer (VSM) at room temperature. As it was seen in Fig. S5, the value of the saturation magnetization was 67 emu/g for the Cu(II) species and magnetic nanoparticles decorated melamine-functionalized chitosan (**1**, See Electronic Supplementary Information).

**Figure 4:**
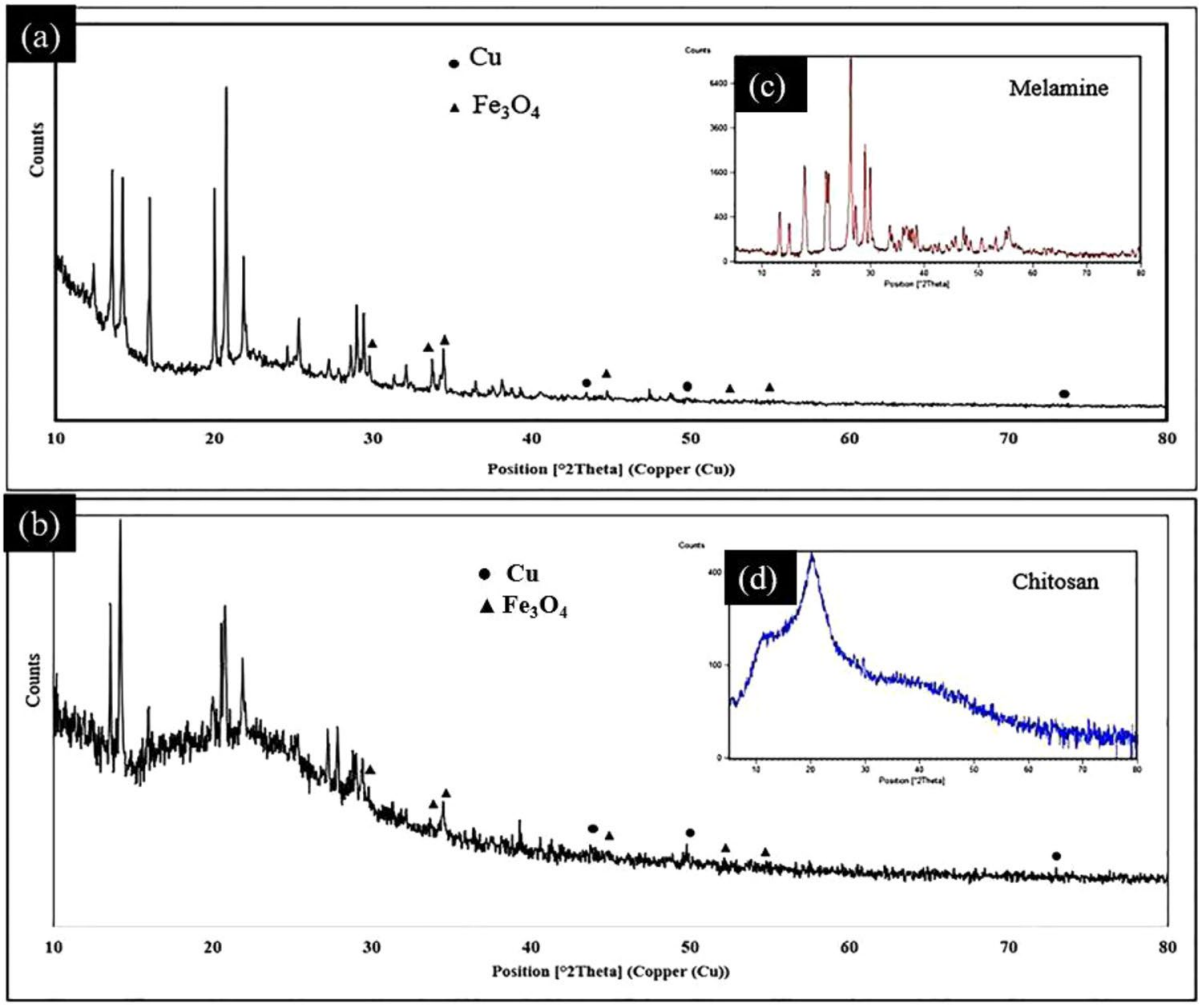
XRD patterns of the fresh Cs-Pr-Me-Cu(II)-Fe_3_O_4_ materials (**1**, **a**), recycled sample after five runs (**b**), melamine (**c**) and commercial chitosan (**d**).

Also, Fig. 4 shows the XRD patterns of the fresh Cs-Pr-Me-Cu(II)-Fe_3_O_4_ (**1**) materials and the recycled sample. The XRD patterns of the melamine and chitosan are also illustrated for comparison as offset patterns. The diffraction peaks at 2θ values of 43.30, 50.40 and 74.15° can be attributed to the reflections of cubic Cu ((JCPDS No. 04–0836, marked with ●). Moreover, the peaks at 2θ values of 30.20, 35.39, 36.89, 53.31, 56.98, 73.91° can be assigned to the reflections of cubic Fe_3_O_4_ (JCPDS No. 019-0629, marked with ▲). On the other hand, the well-defined high intensity diffraction signals (2θ) at 13.41, 17.95, 21.65, 22.25, 26.28, 28.90 and 29.91° are in accordance with the monoclinic crystal system of melamine (JCPDS no. 024-1654). Furthermore, the lower intensity for the diffraction peaks of Cu and Fe_3_O_4_ may be due to their lower weights compared to that of Cs-Pr-Me support in the structure of nanocomposite **1**.

### Cs-Pr-Me-Cu(II)-Fe_3_O_4_ nanomaterials-promoted cascade oxidation/Knoevenagel condensation for the synthesis of α,β-unsaturated nitriles **4a-f**

**Scheme 2:**
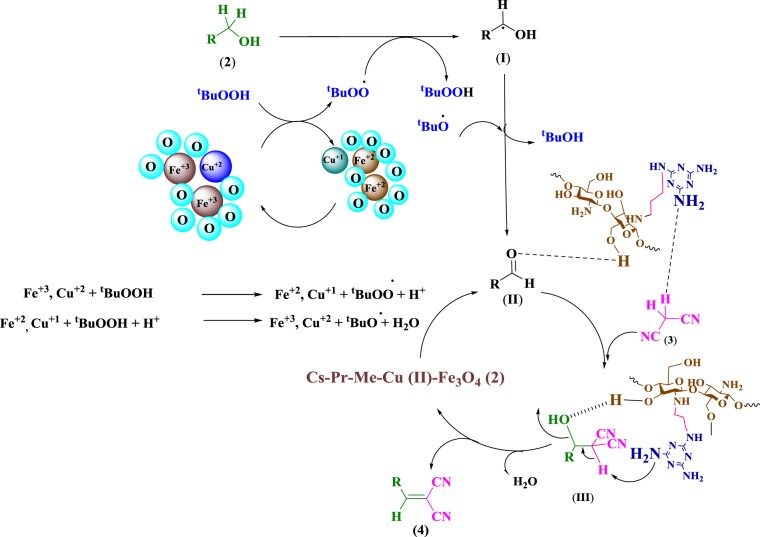
Plausible mechanism for the oxidation/Knoevenagel condensation of different benzyl alcohols **2a-f** catalyzed by Cs-Pr-Me-Cu(II)-Fe_3_O_4_ (**1**).

To evaluate the catalytic activity of Cs-Pr-Me-Cu(II)-Fe_3_O_4_ (**1**), the one-pot oxidation/Knoevenagel condensation between benzyl alcohol (**2a**) and malononitrile (**3**) in CH_3_CN was chosen as the model reaction. As the information in Table 1 show, in the absence of the catalyst **1** and using different oxidants including TBHP, H_2_O_2_, O_2_, and air, no condensation product, 2-benzylidinemalononitrile (**4a**), was formed at 80 °C. However, 58%, 75% and 20% conversion to benzoic acid **6a** was observed when TBHP, H_2_O_2_, and O_2_ were used, respectively. On the other hand, only trace amounts of bezaldehde **5a** and benzoic acid **6a** were formed when the reaction mixture was subjected to an air flow (Table 1, entries 1–4). Interestingly, 23% of the desired benzylidinemalononitrile **4a** was observed by employing 10 mg of Cs-Pr-Me-Cu(II)-Fe_3_O_4_ (**1**), as a catalyst, without any oxidant (Table 1, entry 5). Among TBHP and H_2_O_2_, the former was found to be more effective for the reaction (Table 1, entries 6–7). Indeed, H_2_O_2_ afforded lower yeild of the desired product **4a** compared to TBHP. In fact, the hydroxyl radical produced by H_2_O_2_ is a more powerful oxidant compared to the *t*-BuOO radical generated by TBHP during the reaction. It has been reported before that the OH radical reacts with polysaccharides such as chitosan to depolymerize them and forming chitosan chains with lower molecular weights^57^. Therefore, the lower yeild of the desired product **4a** can be attributed to the higher tendency of OH radicals to depolymerize the chitosan cahins in the structure of the catalyst **1** rather than oxidation of benzyl alcohol (Table 1, entry 7). Hence, TBHP was used in the next optimization experiments. To our delight, by reducing the reaction tempereture, the desired product **4a** was formed in the same yeilds at 60 °C and room tempereture, however longer times were required (entries 8,9). Hence, room temperature was chosen as a sustainable conditions for the reaction although it requires a longer time. Furthermore, the nature of the solvent showed a significant impact on the oxidation of benzyl alcohol (**2a**) and subsequent Knoevenagel condensation. For instance, tolouene and H_2_O afforded the desired product **4a** in lower yields compared to CH_3_CN under the same catalyst loading even after longer times (Table 1, entries 10,11). Upon increasing of the catalyst loading from 5 to 20 mg, the conversion of benzyl alcohol **2a** to 2-benzylidinemalononitrile (**4a**) considerably increased (Table 1, entries 7, 12–14). It is noteworthy that the Cs-Pr-Me-Cu(II) materials afforded a lower yeild compared to the Cs-Pr-Me-Cu(II)-Fe_3_O_4_ (**1**) under the same conditions (entries 14, 15). This can be attributed to the involvement of Fe(III) species in the catalytic cycle (See Scheme 2).

**Table 1:** Screening of different conditions on the oxidation/Knoevenagel condensation products of benzyl alcohol (**2a**)^a^.


Entry	Catalyst	Catalyst loading (mg)	Oxidant	Solvent	Temperature (°C)	Time (h)	Conversion (%) 4a	Conversion (%) 5a	Conversion (%) 6a
1	—	—	TBHP^b^	CH_3_CN	80	24	—	Trace	58
2	—	—	H_2_O_2_	CH_3_CN	80	24	—	Trace	75
3	—	—	O_2_	CH_3_CN	80	24	Trace	Trace	20
4	—	—	Air	CH_3_CN	80	24	—	Trace	Trace
5	Cs-Pr-Me-Cu(II)-Fe_3_O_4_ (**1**)	10	—	CH_3_CN	80	24	23	Trace	—
6	Cs-Pr-Me-Cu(II)-Fe_3_O_4_ (**1**)	10	TBHP^b^	CH_3_CN	80	6	64	—	—
7	Cs-Pr-Me-Cu(II)-Fe_3_O_4_ (**1**)	10	H_2_O_2_^b^	CH_3_CN	80	6	16	trace	27
8	Cs-Pr-Me-Cu(II)-Fe_3_O_4_ (**1**)	10	TBHP^b^	CH_3_CN	60	9	62	—	—
9	Cs-Pr-Me-Cu(II)-Fe_3_O_4_ (**1**)	10	TBHP^b^	CH_3_CN	r.t.	12	62	—	—
10	Cs-Pr-Me-Cu(II)-Fe_3_O_4_ (**1**)	10	TBHP^b^	Toluene	r.t.	15	58	—	—
11	Cs-Pr-Me-Cu(II)-Fe_3_O_4_ (**1**)	10	TBHP^b^	H_2_O	r.t.	24	34	27	—
12	Cs-Pr-Me-Cu(II)-Fe_3_O_4_ (**1**)	5	TBHP^b^	CH_3_CN	r.t.	12	62	—	—
13	Cs-Pr-Me-Cu(II)-Fe_3_O_4_ (**1**)	15	TBHP^b^	CH_3_CN	r.t.	10	87	—	—
14	Cs-Pr-Me-Cu(II)-Fe_3_O_4_ (**1**)	20	TBHP^b^	CH_3_CN	r.t.	8	100	—	—
15	Cs-Pr-Me-Cu(II)	20	TBHP^b^	CH_3_CN	r.t.	10	93	—	—

^a^The model reaction was run at 5 mmol scale. ^b^5 mmol of TBHP or H_2_O_2_ was used for the reaction.

**Figure 5:**
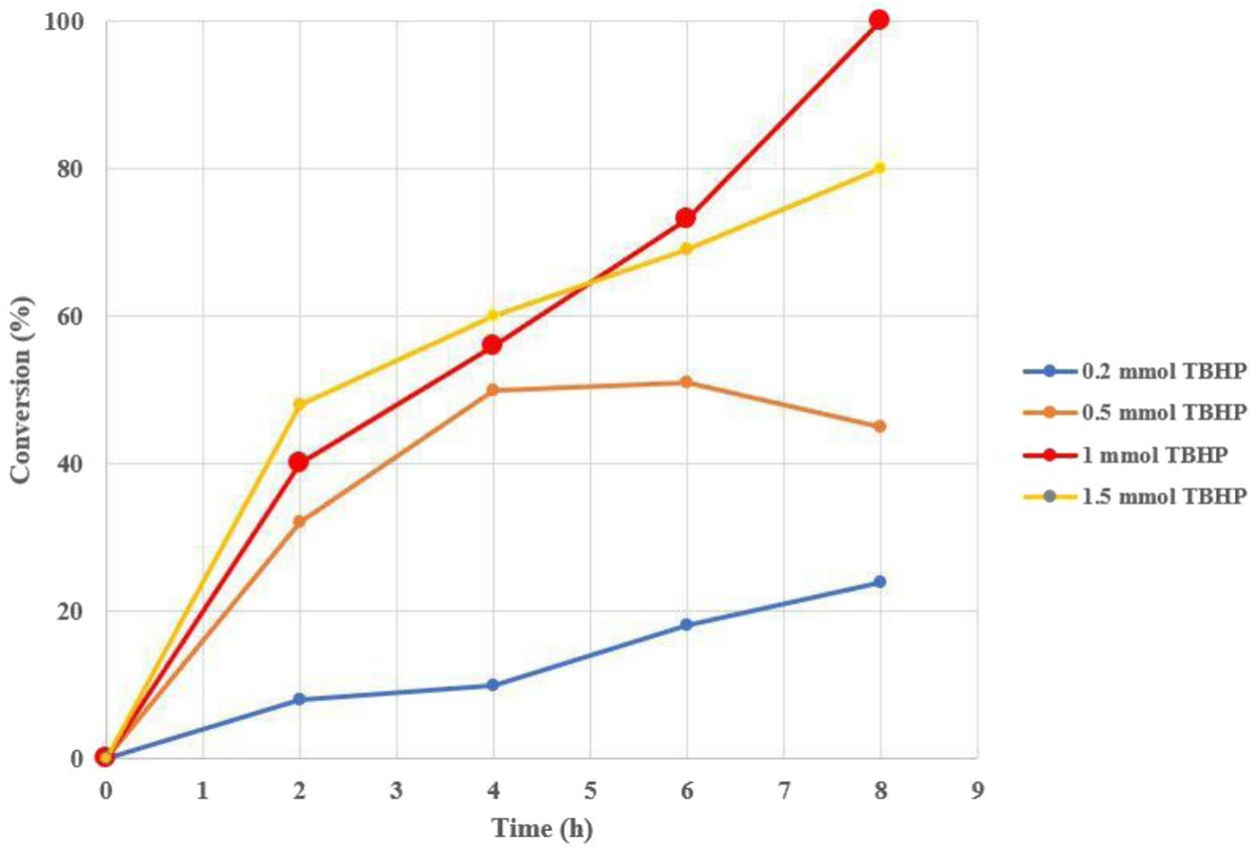
The effect of different TBHP oxidant: benzyl alcohol (**2a**) mole ratios on the yield of oxidation/Knoevenagel condensation product **4a**.

In the next step, the effect of TBHP oxidant equivalent against benzyl alcohol (**2a**) was systemically investigated in 4 h intervals. The results have been shown in Fig. 5. The results of this part of our study showed that one equivalent of TBHP oxidant afforded the desired oxidation/Knoevenagel product **4a** with 100% conversion after 8 h. On the other hand, higher or lower equivalents of TBHP oxidant produced lower yeilds of the desired oxidation/Knoevenagel product **4a**. Encouraged by these results, the substrate scope of this oxidation/Knoevenagel condensation was studied in the next step under the optimal conditions. Table 2 shows the summerized results.

**Table 2:** Scope of the cascade oxidation/Knoevenagel condensation of different benzyl alcohols **2a-f** catalyzed by Cs-Pr-Me-Cu(II)-Fe_3_O_4_ (**1**) under optimized conditions^a,b^.

Entry	Substrate 2	Time (h)	Crude Yield (%)	Conversion (%)		
				4a-f	5a-f	6a-f
1		8	87	100	—	—
2		8	91	79	—	—
3		7	86	51	—	—
4		12	69	88	—	—
5		11	64	67	—	—
6		24^c^	47	—	—	43
		24^d^	95	—	—	90

^a^Reaction conditions: benzyl alcohol derivatives (**2**, 1 mmol), malononitrile (**3**, 1.1 mmol) and the Cs-Pr-Me-Cu(II)-Fe_3_O_4_ (**1**, 20 mg) in CH_3_CN at room temperature. ^b^All products are known and their structures and conversion were established from their ^1^H NMR spectra data and melting points as compared with authentic samples or literature values. ^c^1 mmol of TBHP was used. ^d^2 mmol of TBHP was used.

As revealed in Table 2, the reaction conditions were compatible with both electron-withdrawing and electron-donating substituents at *p*- as well as *o*- positions of the aromatic ring. Intrestingly, 2-benzylidenemalononitrile (**2a**) could be obtained from corresponding substrate in 100% conversion. Furthermore, alcohols such as *p*-hydroxybenzyl alcohol (**2d**) and *p*-nitrobenzyl alcohol (**2e**), reacted slowly to form the corresponding aldehydes in good conversions. On the other hand, 2-pyridylmethanol (**2f**) did not afford oxidation/Knoevenagel condensation product **4f** under optimized reaction conditions. However, it was partially converted to its corresponding carboxylic acid **6f** when two or more equivalents of TBHP was used. This observations can be attributed to fast conversion of substrate **2f** to its corresponding *N*-oxide which rearrange subsequently to the corresponding 2-pyridinecarboxylic acid (**6f**)^58,59^.

According to the above observations, a plausible free radical mechanism, as shown in Scheme 2, can be proposed for the cascade oxidation/Knoevenagel condensation of different benzyl alcohols **2** by TBHP in the presence of Cs-Pr-Me-Cu (II)-Fe_3_O_4_ (**1**). First, THBP is broken down into *t*-butylproxide radical and proton by reduction of Cu^+^^2^ and Fe^+^^3^ ions. Abstraction of a hydrogen radical from benzyl alcohol derivatives **2** affords corresponding benzyl radicals (**I**) which can combine later with *t*-butyloxide radical to form corresponding benzaldehydes (**II**). Next, the obtained aromatic aldehydes and malononitrile (**3**) are activated by the Lewis acidic and basic sites of multifunctional catalyst **1**, respectively via a typical Knoevenagel condensation route. Finally, elimination of one molecule of water affords desired products **4(a-f)**^60,61^. It should be noted that the hygroscopic nature of the chitosan backbone of the supramolecular catalyst **1** can additionally adsorb water molecules on its surface and hence promote smoothly the Knoevenagel condensation^15,51,62^.

**Figure 6:**
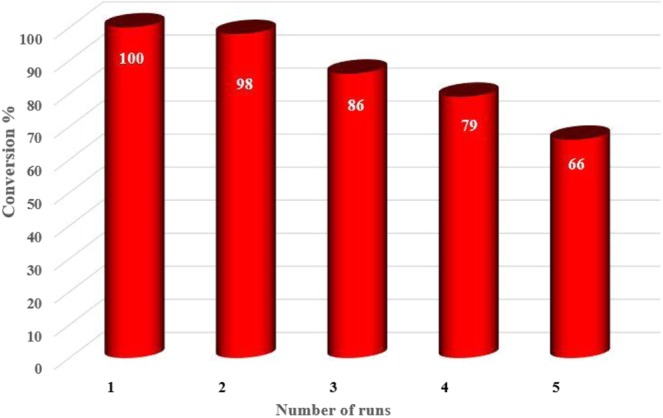
Reusability of the heterogeneous nanocatalyst Cs-Pr-Me-Cu(II)-Fe_3_O_4_ (**1**) for the synthesis of **4a**.

Furthermore, reusability of a heterogeneous catalyst is an important feature for its efficiency and future industrial application. Consequently, we studied the reusability of the Cs-Pr-Me-Cu(II)-Fe_3_O_4_ (**1**) up to fifth cycle (Fig. 6). Therefor, the catalyst **1** was magnetically separated from the reaction mixture, washed with acetone and hexane to remove any organic impurities and dried in an oven. After drying, it was again used for oxidation/Knoevenagel condensation following the same procedure as mentioned in the experimental section. As data in Fig. 6 show, the decrease of catalytic activity of the nanocomposite **1** from the first run to the second run was slight (about 2%). However, more decrease (about 12%) was observed in the next runs with the recycled catalyst **1**. According to ICP analysis results, the percentage of copper and iron in the fresh Cs-Pr-Me-Cu(II)-Fe_3_O_4_ (**1**) was found to be 1.38 wt% (Cu) and 25.72 wt% (Fe), respectively. On the other hand, the percentage of copper and iron in the recycled Cs-Pr-Me-Cu(II)-Fe_3_O_4_ (**1**) after five runs was observed to be 1.26 wt% (Cu) and 25.16 wt% (Fe), respectively. This means that relative percentage of copper loss (8.8 wt%) in the recycled catalyst after five runs is higher than iron loss (2.2 wt%) compared to the fresh sample. These observations can be interpreted to more loss of melamine units, as a more probable chelating agent of Cu(II) species, compared to chitosan monomers with more tendency to chelate Fe_3_O_4_ nanoparticles. Indeed, some parts of the covalent bonds between melamine and chitosan may be broken during mechanical stirring and heating required for reaction or recycling. On the other hand, bridge methylene groups in the 1,3-propylene linker are more labile to be partially oxidised and subsequently facilitate break down of the melamine units from the modified polymer backbone through hydrolysis during reaction, separation or recycling^63^. On the other hand, Fig. 4(b) shows the XRD patterns of the recycled catalyst **1** after five runs used in the model reaction. As can be seen, there is very good coincidenc between the powder XRD signals of the fresh Cs-Pr-Me-Cu(II)-Fe_3_O_4_ (**1**) and the recycled sample.

To demonstrate the efficiency and merits of the Cs-Pr-Me-Cu(II)-Fe_3_O_4_ supramolecular catalyst (**1**) for the cascade oxidation/Knoevenagel condensation of different benzyl alcohols, it has been compared with some of the recently catalytic systems. The comparison has been summarized in Table 3. It is obvious that the present catalytic system requires low loading of a nontoxic and inexpensive transition metal species working at room temperature to afford one-pot oxidation/Knoevenagel condensation products in one step.

**Table 3:** Comparison of the catalytic activity of Cs-Pr-Me-Cu(II)-Fe_3_O_4_ (**1**) with other reported catalysts for the synthesis of the 2-benzylidenemalononitrile (**2a**).

Entry	Catalyst	Catalyst loading	Oxidant	Tempereture (°C)	Time (h)	Conversion (%)	Catalyst reuse times	References
1	Ru(OH)x supported on polyethylenimine modified magnetic nanoparticles coated with silica	100 mg	O_2_	two steps (110 °C + r.t.)	2 steps (10 + 12)	99	2	^64^
2	NiGa Layered Double Hydroxide (CO_3_^-2^@Ni_3_Ga-LDH)	50 mg	O_2_	80 °C	2 steps (4 + 2)	80	5	^45^
3	Gold nanoparticles deposited on an amino-functionalized Al-based MIL-53 metal–organic framework (Au@MIL-53(NH_2_))	1 mol%	O_2_	100 °C	13	99	5	^44^
4	RuCl_3_ on MOF UiO-66 (UiO-66−Ru)	3.6 mol % Ru	(3.6 mol % Ru)	100 °C	6	100	—	^34^
4	Cs-Pr-Me-Cu(II)-Fe_3_O_4_ (**1**)	20 mg	TBHP	r.t	8	100	4	This work

## Experimental Section

### General Information

All reagents and solvents were obtained from commercial suppliers and used without further purification. Chitosan (MW = 100000–300000 Da) was purchased from Acros Organics. ^1^H NMR spectra were recorded at 500 MHz using a Bruker DRX-500 Avance spectrometers in DMSO-*d6* or CDCl_3_ as the solvent. Characterization of the catalyst **1** was carried out using FESEM TESCAN-MIRA3, EDX Numerix DXP-X10P, VSM (BHV-55, Riken, Japan), Shimadzu FT-IR-8400S and TEM Philips CM30. The analytical thin layer chromatography (TLC) experiments were performed using Merck 0.2 mm silica gel 60 F-254 Al-plates.

### General procedure for preparation of the Cs-Pr-Me-Cu(II)-Fe_3_O_4_ (**1**)

The melamine-functionalized chitosan (Cs-Pr-Me) was first prepared according to the procudure described in our previous works^51,52^. Next, the Cs-Pr-Me (1.0 g) was suspended in 50 mL of distilled water. To this suspension, Cu(OAc)_2_ (0.5 g) was added and stirring was continued for 12 h. The final dispersed solution was centrifuged and the obtained solid was dried under vacuum for 1 h. Then, Fe_3_O_4_ nanoparticles were fabricated by *in-situ* coprecipitation as follows: Iron(III) chloride hexahydrate (4.6 g, 0.017 mol) and iron(II) chloride tetrahydrate (2.2 g, 0.011 mol) were dissolved in distilled water. The prepared Cs-Pr-Me-Cu(II) was then added into the obtained aqueous solution and heated to 50 °C under N_2_ atmosphere. Then, 25% aqueous ammonia (10 mL) was slowly added to the obtained mixture under vigorous stirring. After 30 min, the precipitate was collected from the solution by an external magnet and washed three times with distilled water (3 × 5 mL). Finally, the obtained brown solid was dried in an oven at 60 °C for 2 h before using.

### Typical procedure for the synthesis of α,β-unsaturated nitriles (**4a-f**) through cascade oxidation/Knoevenagel condensation catalyzed by the Cs-Pr-Me-Cu(II)-Fe_3_O_4_ (**1**)

In a round-bottomed flask, benzyl alcohol (**2**, 1.0 mmol), TBHP (1.0 mmol) and Cs-Pr-Me-Cu(II)-Fe_3_O_4_ (**1**, 20 mg) were mixed in CH_3_CN (2.0 mL) and stirred at room temperature. Then, malononitrile (**3**, 1.1 mmol) was added to the reaction mixture and the mixture was stirred for the appropriate times reported in Table 2. After completion of the reaction, the solvent was evaporated. Then, EtOAc (3 mL) was added to the mixture and the catalyst **1** was separated by an external magnet. Afterwards, n-hexane was added drop wise into the solution untill benzylidinemalononitriles **4** were completely precipitated. The obtained mixture was filtered off and the precipitate were washed with n-hexane and then dried in an oven at 70 °C for 1 h. Alternatively, the products were extracted by EtOAc and the crude reaction mixture after evaporation of the solvent was analyzed by ^1^H NMR (Figs. S6–S10, See Electronic Supplementary Information). The recycled catalyst **1** was washed with acetone and hexane (1 mL), respectively and then dried at 50 °C for 2 h and stored for another run.

### Leaching test of the Cs-Pr-Me-Cu(II)-Fe_3_O_4_ (**1**)

The Cs-Pr-Me-Cu(II)-Fe_3_O_4_ (**1**) was separated from the model reaction mixture after 4 h. The reaction was continued with the filtrate in the absence of nanocatalyst **1** for an extra 4 h. No further increase in the conversion of benzyl alcohol (**2a**) was observed, which approves the catalytically active sites for this oxidation/Knoevenagel condensation are located on the Cs-Pr-Me-Cu(II)-Fe_3_O_4_ (**1**) and do not disperse into the reaction mixture.

## Conclusions

In summary, we have developed a cost-effective and practical route for fabrication of uniform Cs-Pr-Me-Cu(II)-Fe_3_O_4_ nanomaterials for the first time. The new Cs-Pr-Me-Cu(II)-Fe_3_O_4_ supramolecular catalyst was found to be, a valuable magnetically reusable catalyst for sustainable cascade oxidation/Knoevenagel condensation of benzyl alcohols to their corresponding α,β-unsaturated nitriles under truely mild reaction conditions. Significant advantages of the present work are as follows: high to quantitative conversion of benzyl alcohols, mild reaction conditions, scalable synthesis, simple separation of the catalyst using an external magnet and reusability of the catalyst for at least five cycles. Based on this points, the present work can be considered as a valuable complementary study in the field of cascade oxidation/Knoevenagel condensation. Furthermore, this supramolecular catalyst is a successful example of the combination of chemo- and bio-drived materials catalysis for mimicing biocatalysis and sustainable and one pot multistep synthesis.

## Supplementary information

Supplementary information
