# Supplementary material for: Cu(II) and magnetite nanoparticles decorated melamine-functionalized chitosan: A synergistic multifunctional catalyst for sustainable cascade oxidation of benzyl alcohols/Knoevenagel condensation
Source: Sci Rep. 2019 Nov 28;9:17758. doi: 10.1038/s41598-019-53765-3 (PMC6883033; doi:10.1038/s41598-019-53765-3)
Supplement: Supplementary file 1 — Supplementary information [file 41598_2019_53765_MOESM1_ESM.doc]

**Supporting information**

**Cu(II) and magnetite nanoparticles decorated** **melamine-functionalized chitosan: A synergistic multifunctional catalyst for sustainable cascade** **oxidation of benzyl alcohols/ Knoevenagel condensation**

Zahra Alirezvani, Mohammad G. Dekamin*, Ehsan Valiey

*Pharmaceutical and Heterocyclic Compounds Research Laboratory, Department of Chemistry, Iran University of Science and Technology, Tehran, 16846-13114, Iran.*

**E-mail: mdekamin*[*@iust.ac.ir*](mailto:maleki@iust.ac.ir)

| **Page** | **Content** |
| --- | --- |
| S1 | Title page |
| S2 | General procedure for preparation of the Cs-Pr-Me-Cu(II)-Fe_3_O_4_ (**1**) |
| S2 | Typical procedure for synthesis of α, β-unsaturated nitriles through oxidation/ Knoevenagel condensation catalyzed by the Cs-Pr-Me-Cu(II)-Fe_3_O_4_ (**1**) |
| S4 | Characterization of the Cu(II) species on melamine-functionalized chitosan decorated with magnetic nanoparticles (Cs-Pr-Me-Cu(II)-Fe_3_O_4_, **1**) |
| S9 | ^1^H NMR spectrum of the product obtained from the benzyl alcohol (**2a**) oxidation/Knoevenagel reaction vessel. |
| S10 | ^1^H NMR spectrum of the product obtained from the 2-chlorobenzyl alcohol (**2b**) oxidation/Knoevenagel reaction vessel. |
| S11 | ^1^H NMR spectrum of the product obtained from the 4-methoxybenzyl alcohol (**2c**) oxidation/Knoevenagel reaction vessel. |
| S12 | ^1^H NMR spectrum of the product obtained from the 4-hydroxybenzyl alcohol (**2d**) oxidation/Knoevenagel reaction vessel. |
| S13 | ^1^H NMR spectrum of the product obtained from the 4-nitrobenzyl alcohol (**2e**) oxidation/Knoevenagel reaction vessel. |

***General procedure for preparation of*** ***the*** ***Cs-Pr-Me-Cu(II)-Fe_3_O_4_ (1)***

The melamine-functionalized chitosan (Cs-Pr-Me) was first prepared according to the procudure described in our previous works. Next, the Cs-Pr-Me (1.0 g) was suspended in 50 mL of distilled water. To this suspension, Cu(OAc)2 (0.5 g) was added, and stirring was continued for 12 h. The final dispersed solution was centrifuged and the obtained solid was dried under vacuum for 1 h. Then, Fe3O4 nanoparticles were fabricated by in-situ coprecipitation as follows: Iron(III) chloride hexahydrate (4.6 g, 0.017 mol) and iron(II) chloride tetrahydrate (2.2 g, 0.011mol) were dissolved in distilled water. The prepared Cs-Pr-Me-Cu(II) was then added into the obtained aqueous solution and heated to 50 ºC under N_2_ atmosphere. Then, 25% aqueous ammonia (10 mL) was slowly added to the obtained mixture under vigorous stirring. After 30 min, the precipitate was collected from the solution by an external magnet and washed three times with distilled water (3 × 5 mL). Finally, the obtained brown solid was dried in an oven at 60 oC for 2h before using.

***Typical procedure for synthesis of α, β-unsaturated nitriles* *through oxidation/ Knoevenagel condensation catalyzed by the*** ***Cs-Pr-Me-Cu(II)-Fe_3_O_4_ (1)***

In a round-bottomed flask, benzyl alcohol (**2**, 1.0 mmol), TBHP (1.0 mmol) and Cs-Pr-Me-Cu(II)-Fe_3_O_4_ (**1**, 20 mg) were mixed in CH_3_CN (2.0 mL) and stirred at room tempereture. Then, malononitrile (**3**, 1.1 mmol) was added to the reaction mixture and the mixture was stirred for the appropriate times reported in Table 2. After completion of the reaction, the solvent was evaporated. Then, EtOAc (3 mL) was added to the mixture and the catalyst **1** was separated by an external magnet. Afterwards, n-hexane was added drop wise into the solution untill benzylidinemalononitriles **4** were completely precipitated. The obtained mixture was filtered off and the precipitate were washed with n-hexane and then dried in an oven at 70 ^o^C for 1 h. Alternatively, the products were extracted by EtOAc and the crude reaction mixture after evaporation of the solvent was analyzed by ^1^H NMR. The recycled catalyst **1** was washed with acetone and hexane (1 mL), respectively and then dried at 50 °C for 2 h and stored for another run.

**Scheme S1**. Oxidation/ Knoevenagel condensation of different benzyl alcohol derivatives with with malononitrile in the presence of the Cs-Pr-Me-Cu(II)-Fe_3_O_4_, **1**)..

***Characterization of the Cu(II) species on melamine-functionalized chitosan decorated with magnetic nanoparticles (Cs-Pr-Me-Cu(II)-Fe_3_O_4_, 1).***


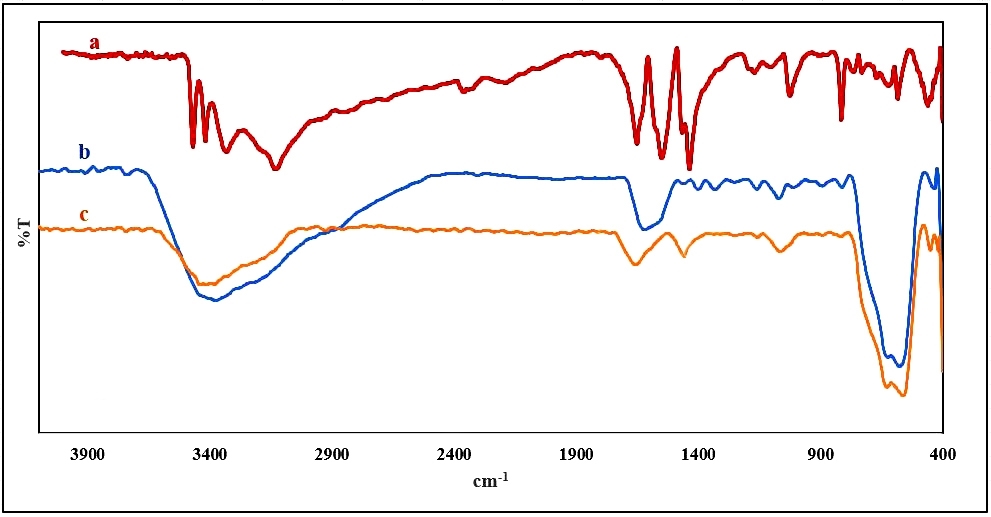


**Figure S1.** FTIR spectra of the Cs-Pr-Me before modification (a), Cs-Pr-Me-Cu(II)-Fe_3_O_4_, **1**) (b) and the Cs-Pr-Me-Cu(II)-Fe_3_O_4_ **(1)** after reused five times.


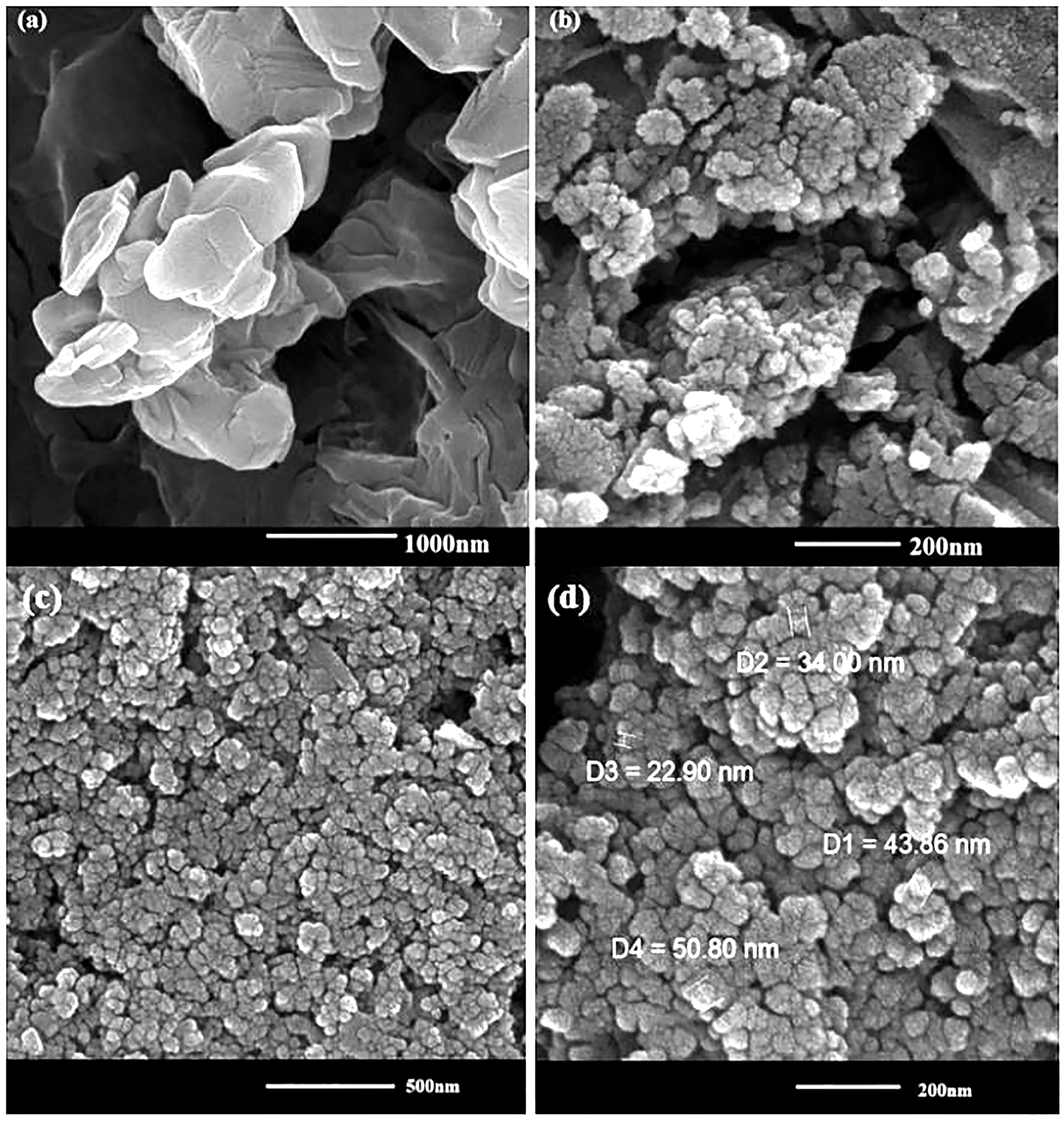


**Figure S2.** FESEM images of the commercial chitosan (a), the Cs-Pr-Me befor modification (b), and Cs-Pr-Me-Cu(II)-Fe_3_O_4_, **1**) at 500 (c) and 200 nm (d) scales.


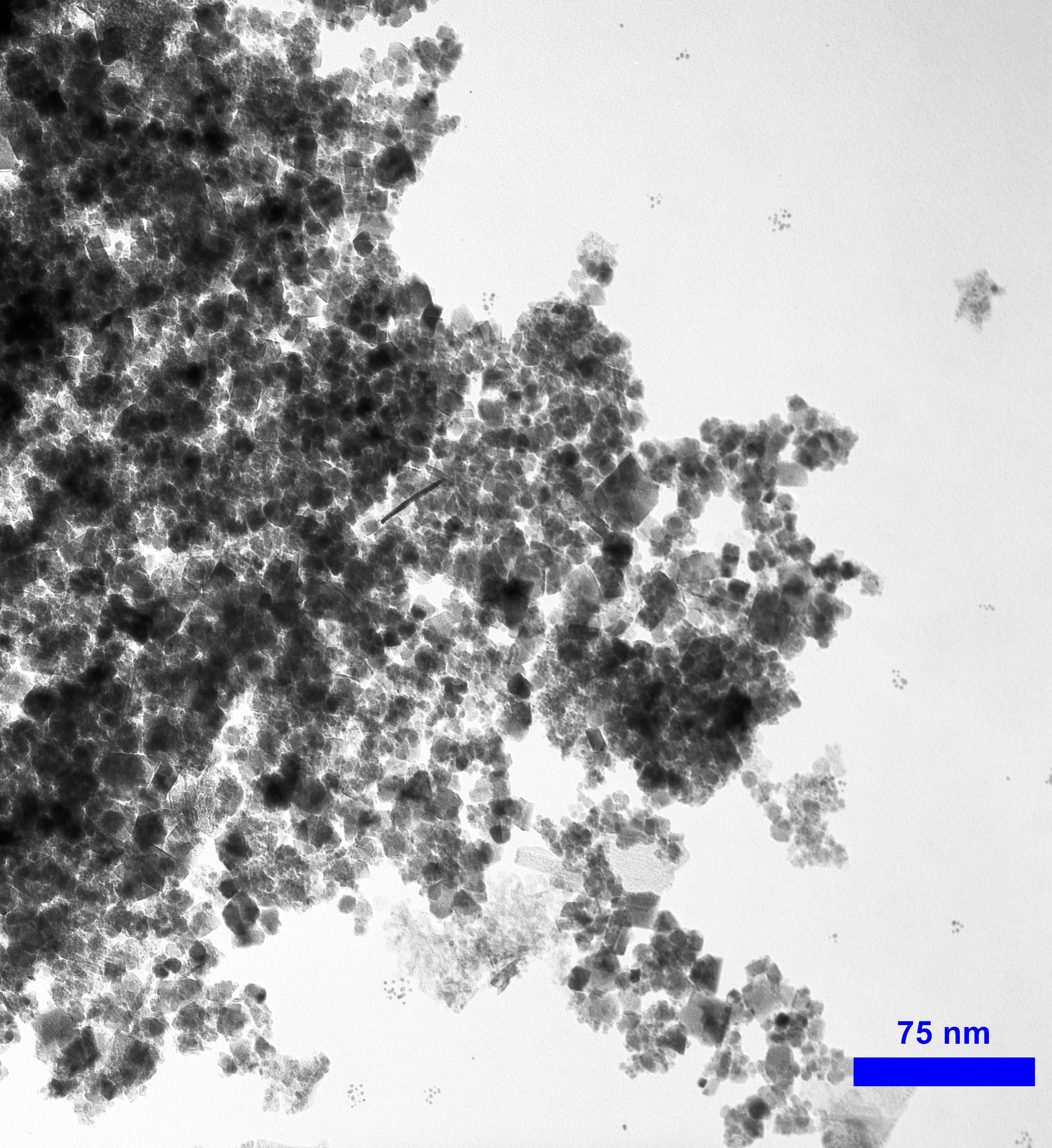


**Figure S3.** TEM images of the Cs-Pr-Me-Cu(II)-Fe_3_O_4_ (**1**)**.**


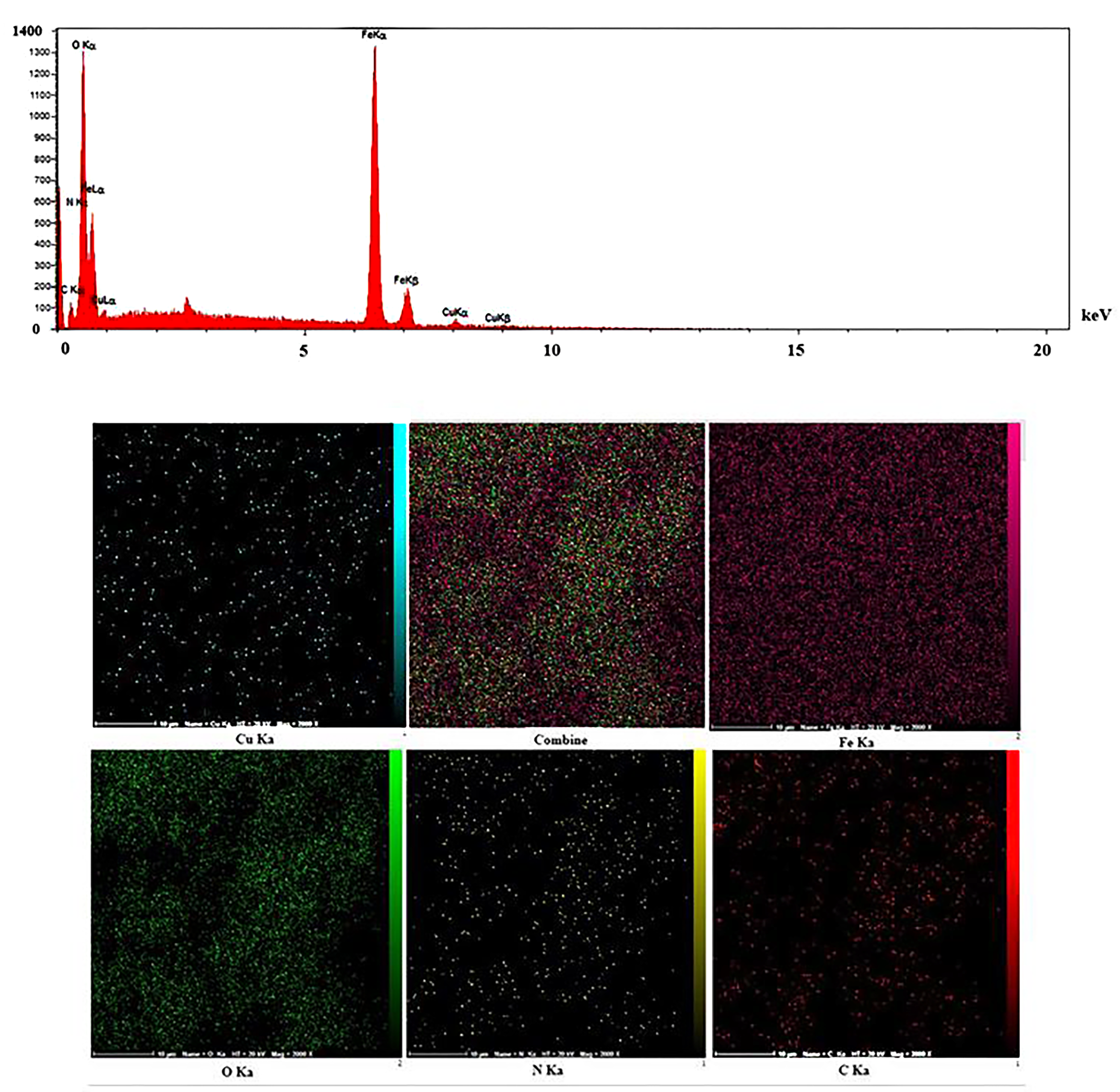


**Figure S4.** Energy dispersive spectroscopy (EDX) pattern and elemental mapping of the Cs-Pr-Me-Cu(II)-Fe_3_O_4_ (**1**)**.**


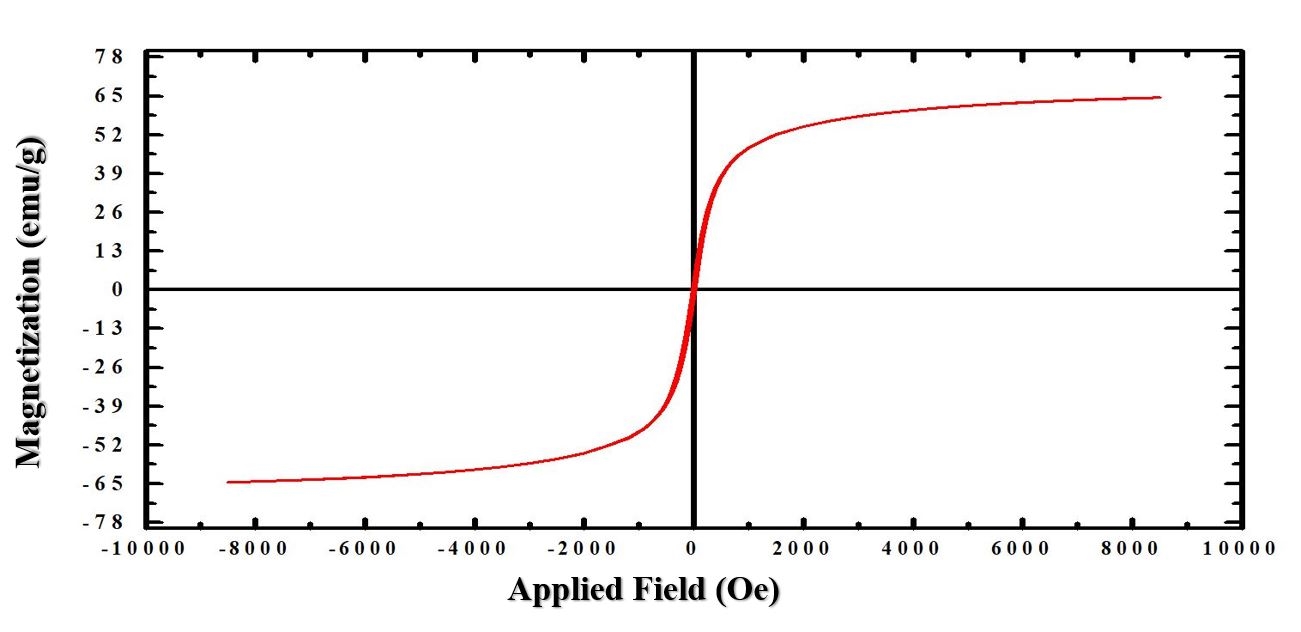


**Figure S5.** VSM magnetization curve of the Cs-Pr-Me-Cu(II)-Fe_3_O_4_ (**1**)**.**


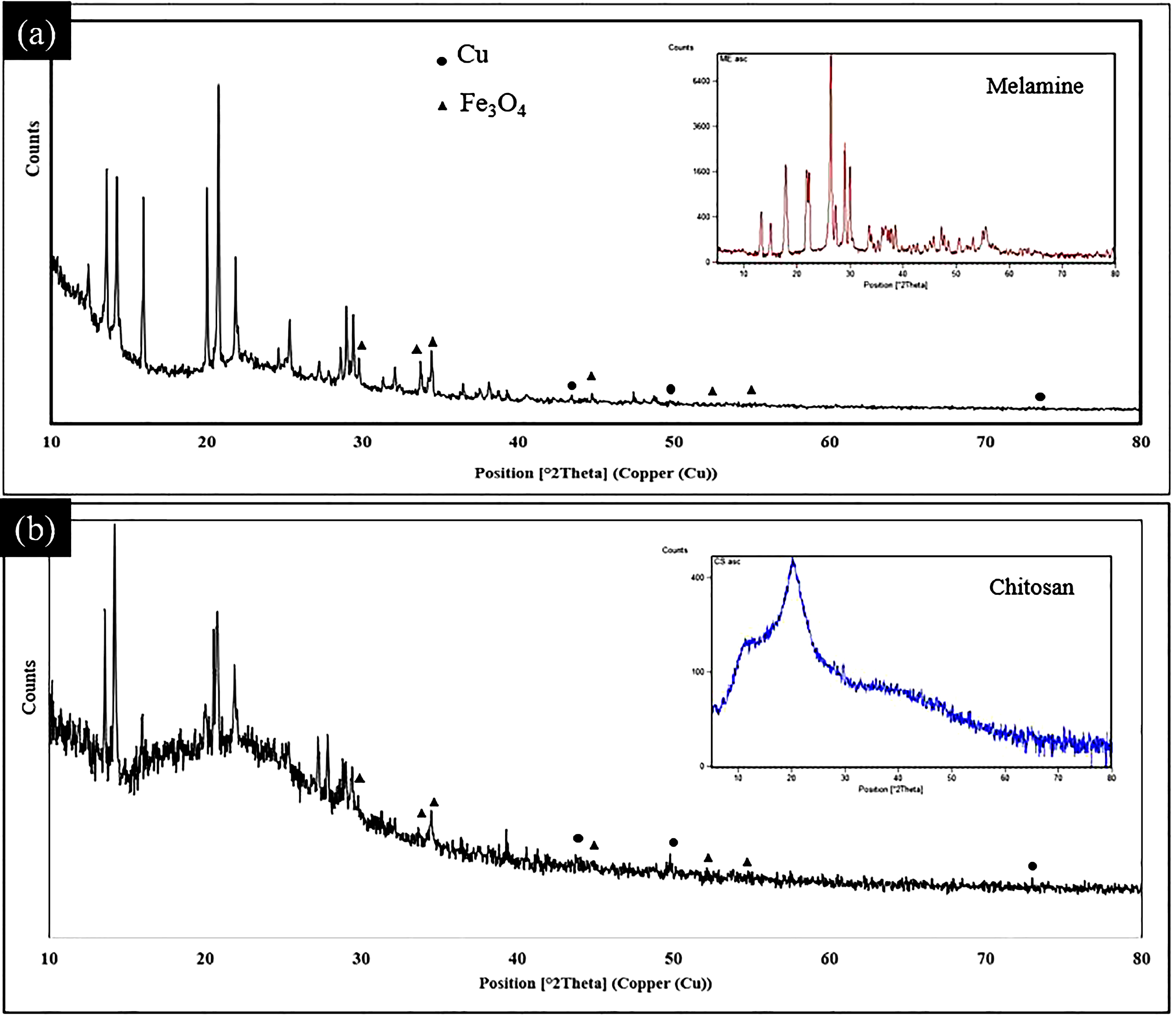


**Figure S6.** The XRD pattern of a) the Cs-Pr-Me-Cu(II)-Fe_3_O_4_ (**1**) and b) the Cs-Pr-Me-Cu(II)-Fe_3_O_4_ after five time using in model reaction**.**


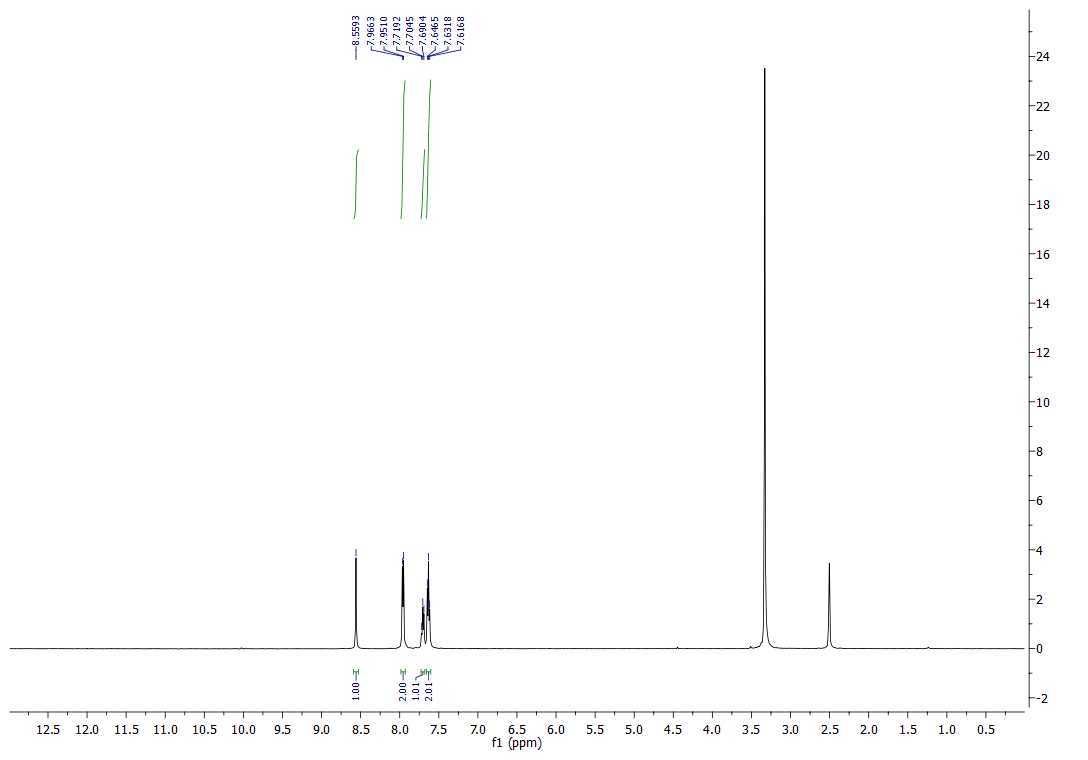


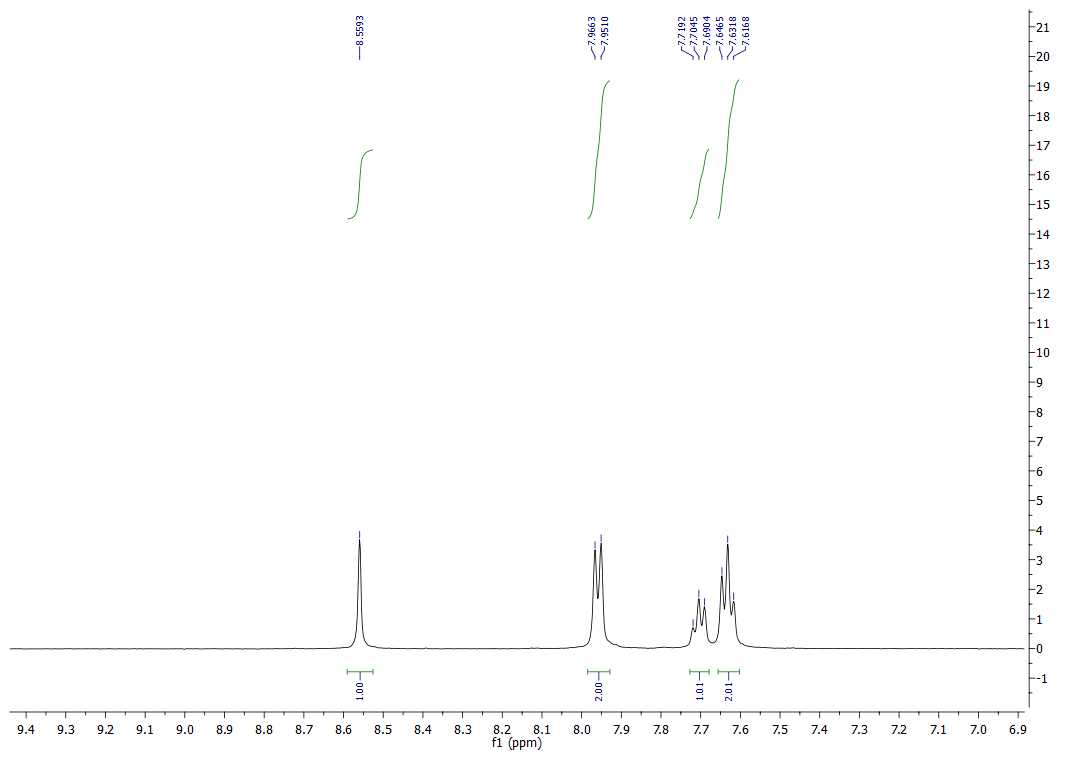


**Figure. S5.** ^1^H NMR spectrum of the product obtained from the benzyl alcohol (**2a**) oxidation/Knoevenagel reaction vessel.


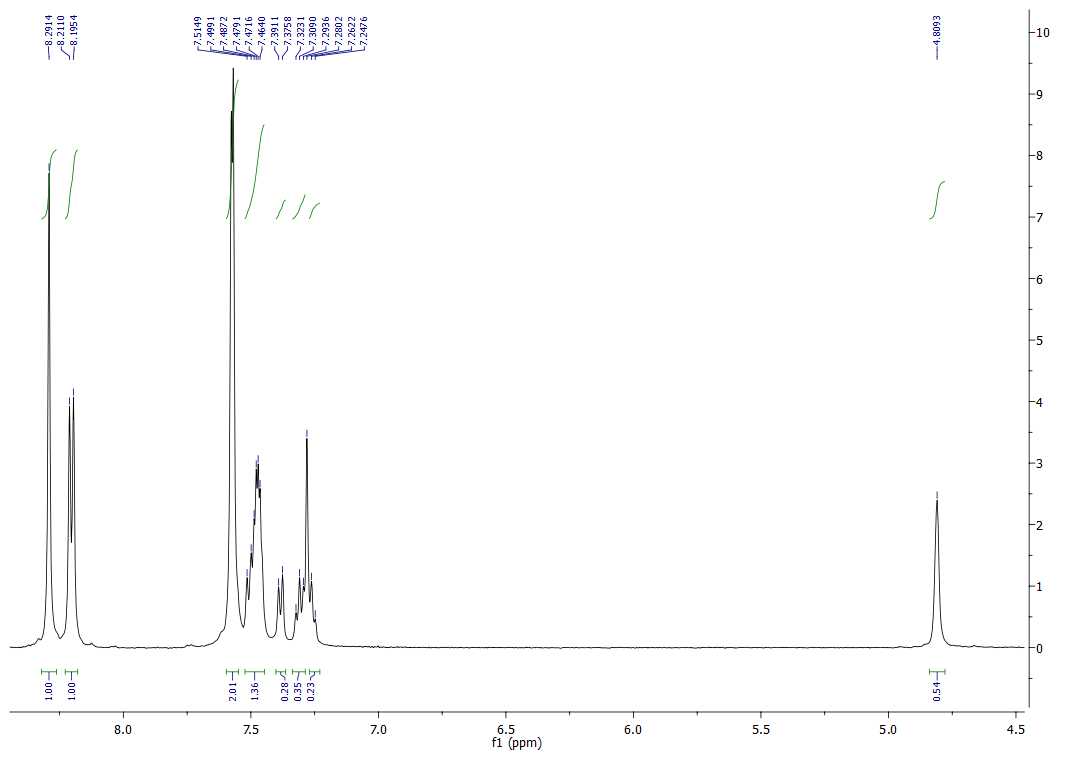


**Figure. S6.** ^1^H NMR spectrum of the product obtained from the 2-chlorobenzyl alcohol (**2b**) oxidation/Knoevenagel reaction vessel.


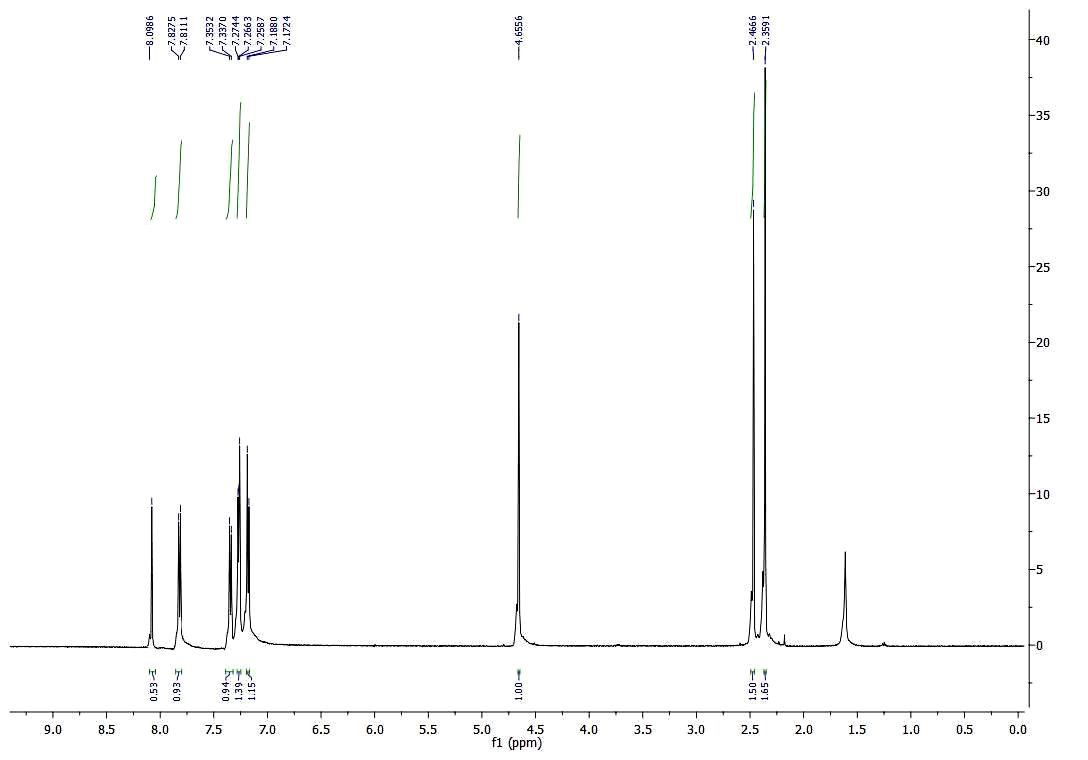


**Figure. S7.** ^1^H NMR spectrum of the product obtained from the 4-methoxybenzyl alcohol (**2c**) oxidation/Knoevenagel reaction vessel.


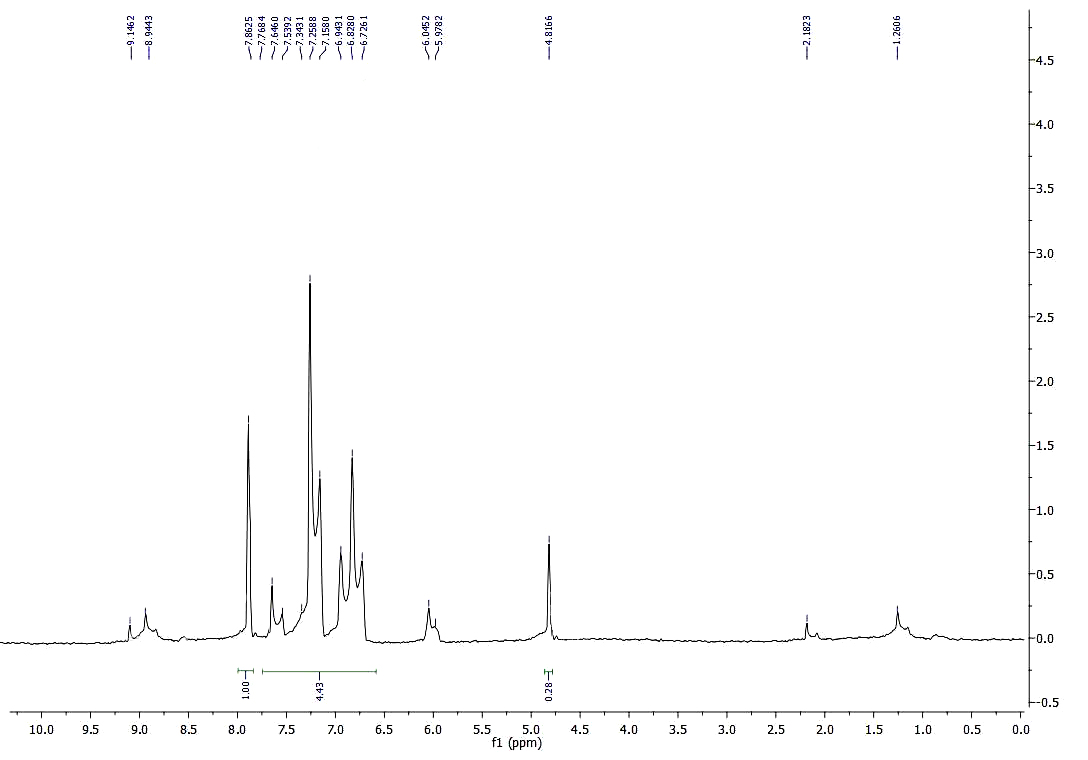


**Figure. S8.** ^1^H NMR spectrum of the product obtained from the 4-hydroxybenzyl alcohol (**2d**) oxidation/Knoevenagel reaction vessel.


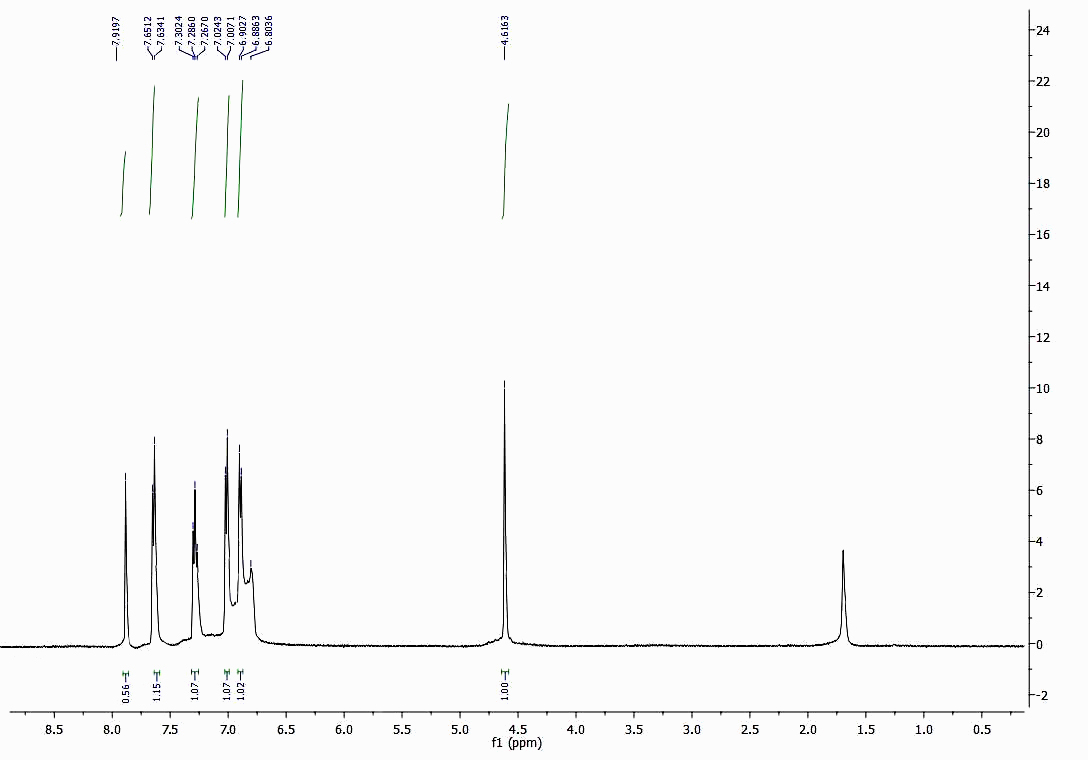


**Figure. S9.** ^1^H NMR spectrum of the product obtained from the 4-nitrobenzyl alcohol (**2e**) oxidation/Knoevenagel reaction vessel.
